# Supervised practice program guided by the Accreditation Council for Education in Nutrition and Dietetics standards improves potential employability of nutrition/dietetics graduates: perspective of employers and preceptors

**DOI:** 10.1186/s12909-019-1893-3

**Published:** 2019-12-10

**Authors:** Hiba Bawadi, Ghadir Fakhri Al-Jayyousi, Xiangyun Du, Vijay Ganji, Abdelhamid Kerkadi, Joyce Moawad, Taghreed Abunada

**Affiliations:** 1 Human Nutrition Department, College of Health Science, QU Health, Qatar University, Doha, Qatar; 2 Department of Public Health, College of Health Science, QU Health, Qatar University, Doha, Qatar; 3 College of education, Qatar University, Doha, Qatar; 4 Department of Biomedical Sciences, College of Health Sciences, QU Health, Qatar University, Doha, Qatar

**Keywords:** Dietetics education, Supervised practice program, Employment, Internship, International accreditation standards, Accreditation Council for Education in dietetics and nutrition, ACEND

## Abstract

**Background:**

This study investigated employers’ perspectives on the impact of a supervised practice program (SPP), guided by international accreditation standards on the skill development and potential employability of dietetics students.

**Main body:**

This study was based on qualitative research. Fifteen potential employers, who also served as SPP preceptors, participated in this study. Participants were interviewed using semi-structured questionnaire. All interviews were conducted face-to-face by a trained interviewer. Participates were invited to discuss their own experiences in the current SPP, their perceptions of the impact of SPP on skills and attributes of graduates, and their overall ideas of how SPP may contribute to the employability of graduates.

This study found that the SPP program guided by international accreditation standards shaped the duties and responsibilities of preceptors/supervisors and promoted a beneficial relationship between preceptors and SPP students. The benefits to graduates include bridging the gap between classroom didactic knowledge and practice; improving workplace self-confidence; developing competencies such as critical-thinking, communication, interviewing, and counselling skills in various multidisciplinary and multicultural settings. In addition, the preceptors suggested that accreditation-guided SPP contributes to the employability of graduates. Also, they opined that it reduced the need for orientation and shortened the probation time because students were familiar with the work environment and work flow. This lead to the improved preparedness for work.

**Conclusion:**

SPP based on set of competencies guided by international accreditation standards provides an up-to-date curriculum, improves the quality of the nutrition and dietetics services, and increases the potential employability of the graduates.

## Background

Qatar University’s Human Nutrition Program (HNP) is one of the five international programs accredited by the USA’s Accreditation Council for Education in Nutrition and Dietetics (ACEND) of Academy of Nutrition and Dietetics [1]. For international programs to be accredited, the programs should meet the most recent standards set by the ACEND. A major component of ACEND accredited programs is the Supervised Practice Program (SPP) (internship). SPP involves the integration of academic knowledge and skills with practical experience to develop specific competencies through repetition [2]. After a successful completion of SPP, students must pass the registration examination to obtain practice license. These licensed graduates are called Registered Dietitians (RD) and are considered competent for entry-level dietitian/nutritionist positions. Several studies have documented the importance of SPP in healthcare education and employability [2–5]. Participants’ responses indicated that SPP was a critical aspect of dietetics education for the development of competencies, confidence, knowledge, and skills to practice as an RD [2]. A study in Iran reported that one of the problems in the health care education was the translation of theory-based knowledge into practice in various clinical settings [5].

It is known that lack of employability opportunities is a concern for both graduates and health professional program administrators. Employability represents the transition from formal education to the workforce. It is defined as either the ability to obtain employment after graduation or as a range of skills that students need to master in order to become employable [6, 7]. Although several studies have documented the importance of experiential education in employability [2, 8, 9], there is a paucity of data on the impact of SPP on the preparation for employment from an employer’s perspective in an international dietetic education setting [9].

There are a total of 5 international nutrition/dietetics programs (UAE, Lebanon, Qatar, Mexico, and South Korea) are accredited by the ACEND. HNP program at Qatar University has been approved to take 30 students per academic year [1]. All these 30 students must undergo didactic education for 3.5 years and SPP for 1 year [10]. After completion of SPP and obtaining RD credentials, students seek employment in hospitals in clinical setting and non-clinical setting, foodservice industry, sports facilities, food industry, and public health area [11]. The impact of an international dietetics accreditation on the development of skills and attributes and on the overall potential employability of graduates has never been studied in an internationally accredited program. Therefore, the objective of this study was to investigate the impact of a SPP guided by international dietetics accreditation standards (IDAS) of ACEND on the employability of dietetics/nutrition graduates.

## Methods

### Setting

The HNP at Qatar University offers didactic coursework and 1200 h of mandatory SPP for dietetics students. Students enter the SPP after completion of 3.5 years of didactic coursework. SPP students are assigned to one supervisor at each site and rotated through several primary, secondary, and tertiary healthcare facilities in Doha city area. Because Qatar University does not have a teaching hospital yet, we used local health care facilities to train our SPP students. We have formal contracts in place to train 30 Qatar University SPP students in various health care facilities in the Doha region.

### Participants

A purposive technique was employed for sampling. Although our SPP program has about 50 preceptors in various healthcare facilities, only 15 of them had the staff recruiting authority. Therefore, we selected 15 preceptors/supervisors to participate in this study. These preceptors were not employed by the Qatar University. An open email was sent to all supervisors of SPP program (*n* = 15) to participate in this study. None refused to participate in the study. All supervisors had prior precepting experience in the SPP program for dietetics students from Qatar University. Fifteen participants (11 women; 4 men) volunteered to participate in this study. The participants were employed for about 5–15 years as dietitian in various health care facilities in Qatar. Out of 15 participants, 5, 4, 3, and 3 obtained RD credentials from India, Philippines, Qatar, and Europe, respectively. These were dietitians with administrative job responsibilities with decision making role in recruitment and hiring of dietitians. The majority of the participants (12 out of 15) had precepting experience not only in the Qatar University SPP but also in other academic institutions as preceptors. Participants in this study held leadership positions in different dietetics fields. Positions held by these participants were director/head of dietetics department, clinical dietetics supervisor, dietetics service supervisor, pediatric dietetics supervisor, and supervisor of dietitians of oncology and home healthcare services. Each supervisor precepts 1–3 SPP students at any given time.

### Study design and data collection

This is a qualitative study with a cross-sectional design. The qualitative research design was used because this allowed us to perform in depth analysis of study participants’ attitudes, behaviors, and perceptions. Also this design encouraged participants to be open in their responses. This study was conducted employing semi-structured interviews as the major data-generation method. These type of interviews were aimed to explore individuals’ experiences, opinions, and insights [12]. A control-group was not considered because all HNP students must undergo SPP after completion of academic course work at Qatar University. Hence, we do not have HNP program without SPP. The written consent form and all study protocols were approved by the Institutional Review Board at Qatar University. Data collection was conducted between August 15, 2018 to April 30, 2019. Prior to the interviews, purpose of the research, methods, and potential contribution to the SPP program were explained to all participants. Participants were ensured confidentiality and the right to withdraw from the study. Only after the participants signed the written consent form, we conducted semi-structured face-to-face interviews. The preceptors were invited to discuss their own experiences in the current SPP program, and their perceptions of the impact of SPP on graduates’ skills and attributes. They were also invited to discuss their overall ideas of how SPP may have contributed to the employability of graduates. All interviews were conducted in English. Each interview lasted approximately 30–40 min and was digitally audio-recorded and later transcribed.

### Data analysis

The interview analysis process had several phases. Firstly, the authors individually read through the transcripts using an inductive content-analysis technique to organize individual responses by identifying communication patterns and exploring meanings via linguistic features in their given contexts [13]. Then we worked independently to analyze the data and identify common themes. In the process of the thematic analysis, we focused on meaning condensation by outlining the meanings expressed by the preceptors through coding and categorizing into shorter formulations [12]. Following constant comparative techniques [14], each transcript was coded, and new themes were added to the codebook as they emerged. Afterwards, we discussed the results and patterns and worked together until consensus was reached on interpretation of the patterns. The rounds of discussion were also an attempt to overcome our potential bias and improve our internal reliability [13]. Then we sent an agreed version of the analysis of results to the participants for verification of the material [12]. We received no objection but a few additional information and explanations were added to the final version of the results. In the process of analysis, specific attention was given to contextual analysis in order to gain insights of the cultural meanings of the interviews.

## Results

The analysis of the interviews revealed several themes. These were (1) employers and preceptors’ own experiences in an IDAS-guided SPP program, (2) employers and preceptors’ perspectives on how SPP affected the development of skills and attributes for graduate students, (3) employers and preceptors’ perspectives of how SPP may have contributed to potential employability, and (4) other factors that contribute to employability.

### Employers and preceptors’ own experiences in an IDAS-guided SPP program

Almost all participants (*n* = 14) reported that SPP shaped their roles while supervising/precepting students at the training site. They (*n* = 6) explained that they always provided students with up-to-date, evidence-based information. Supervisors stated that they received annual refreshment courses on preceptorship skills. One supervisor revealed that “all information given to the students was evidence-based and this was one of the important roles of clinical dietitian”. Participants felt that they needed to update themselves through reviewing current articles and attending professional meetings. They felt that these continuous education activities are crucial in obtaining the evidence-based information.

Supervisors (*n* = 6) explained that the SPP had specific criteria and clear objectives to meet in order to achieve the competencies. They were familiar with the core skills and competencies students should acquire from their SPP experience. Supervisors were also clear on students weekly and daily evaluation/feedback on their SPP rotation. Three participants explained that the SPP program helped them recognize their weaknesses and strengths as supervisors, improve their practice, improve their knowledge, and improve their ability to help students complete the practical experience. A few supervisors (*n* = 4) pointed that the preceptor training helped them foster professional relationship with their students. They felt that this close professional rapport with their trainees facilitated the communication. This improved communication allowed SPP students to get their questions answered in a timely manner, receive specific advice and direction, and receive feedback to improve their performance.

### Employers and preceptors’ perspectives on how SPP affects the development of skills and attributes for graduate students

#### Connect theoretical knowledge to practice

The second theme emerged from this study was the impact of IDAS-guided SPP on students’ development. Participants explained how applying IDAS to the SPP program helped dietetics students acquire the skills and competencies they would gain from an internship and be competent candidates for entry-level RD positions in the global market. All participants felt that the SPP training helped students to connect their theoretical knowledge to real, practical experience, improve their critical-thinking skills, enhance their communication, interviewing and counseling competencies, improve their self-confidence when dealing with real cases, and become multicultural competent dietitians that are able to work within multidisciplinary teams for the benefit of the patient. Some examples cited in this theme were taking dietary history and measurement, counseling and documentation, monitoring and delivering an intervention, following up, and making decisions. One supervisor stated “Every day is a new learning journey for them. They are exposed to huge number of patients to take the dietary history, assess the patient, and interpret the laboratory results in the light of nutrition intake. I think this enhances their learning, and this will help them apply the science of nutrition to the clinical setting. Overall from this practical training students became more adaptable, confident, life-long learners, and innovators”.

#### Improve their critical-thinking skills

Participants (*n* = 9) addressed how this training enhanced students’ critical-thinking skills. Students would follow abstract and propositional ways of thinking when comparing different cases and apply interventions in various situations. One participant mentioned: We usually assess their critical thinking skills and how they bring the evidence to the practice. When we review the patients’ nutrition records, we (preceptors) evaluate PES (Problem, Etiology, and Signs and Symptoms) statements written by SPP students for these patients? As part of the follow up, we ask students to prepare 5–6 PES statements for the same patient and choose the best one that fits the patient’s condition. When they come back, they need to explain how and why. This is critical thinking skills we look for. Everybody can write PES, but the basic skill is critical thinking for which one has the priority that is most suitable for this case.”

#### Enhance their communication, interviewing and counseling skills

The SPP helped students to develop their communication skills with patients, peers and supervisors. According to the participants, students gained the skills crucial for counselling and group teaching. Students would also learn how to interview patients and obtain the rich information needed to put the patient on a specific diet plan and effective interventions. One of the supervisors explained that “active listening” is very crucial skills in counselling. This allows them to formulate an appropriate counselling strategy and feedback. SPP students are taught to emphasize the positive behaviors first and then to focus on ways to improve other dietary issues patients might have. A few supervisors (*n* = 4) talked about how handling a case, being responsible for managing it, planning an effective intervention, and following up with the patient to assess progress would help improve students’ self-confidence. This will make SPP students more confident and competent which make them well prepared for the workforce as professional specialists.

#### Be multicultural and multidisciplinary competent

During their SPP, students would work with patients from different nationalities. They would learn about different cultures and learn how to communicate with multilingual patients at the training sites. In addition, for the benefit of patients, students would work with multidisciplinary teams of professionals. One participant elaborated on this: “We have multi-cultures, so they are already exposed to patients with different cultural backgrounds. They might not like to eat khobz, dates, or something like this and they prefer rice. Some dietitians force certain foods on patients. They need to respect the value, the attitude, the culture, the religious preferences of patients”. According to the participants, SPP students have the advantage of knowing Arabic culture which may lead to cultural competency.

#### Employers and preceptors’ perspectives of how SPP may have contributed to potential employability

The third theme emerged was employers’ and preceptors’ perspectives on SPP contribution to potential employability. All participants mentioned that applying IDAS to SPP enhanced students’ employability. Participants were supervisors who were involved in the recruitment process for different positions in various nutrition departments. Sub-themes addressing the mediating factors shaping the relationship between applying IDAS to the training program and employability were explored in this research study.

#### No need for long job orientation and probation period

Participants (*n* = 8) mentioned that when they hired students who had completed an SPP, they did not need to give them a long orientation. They might end up having 1–2 month orientation as a refreshment and then students would have their own working areas and could start working independently. One supervisor said: “all details are covered in their training, which motivates us to recruit SPP students, not any candidate from outside. Even with the challenging tasks such as nutrition care and documentation processes, I don’t have to struggle with them if I recruit a student from the Qatar University’s SPP. We are able to take them directly into challenging opportunities, and their output is also excellent.” Majority of supervisors (*n* = 10) explained that SPP students would be exposed to the real work environment before having a real job. They would be exposed to all policies, rules, regulations, and the professional way of conducting their work. They would know how to deal with patients from A to Z. “They have the ability to handle the work. Currently, “I am recruiting some staff”. Sometimes they surprised me with their ready-to-work skills. The good thing is that they are confident and they have a very strong background and rich practical experience”. “If I have two candidates, one without and one with SPP, I’ll choose the one with the SPP because the SPP student has more practical knowledge with ready to work skill set”. One participant said that SPP gives the students a clear picture about their future real practice and it equips them with basic skills and competencies about different domains of practice.”

#### Frequently updated curriculum

Participants mentioned that IDAS driven SPP has specific objectives to be achieved and guidelines to be followed. Yet, there was a flexibility in this training program. Supervisors from the training sites and from the academic institution would work together to update the curriculum in order to meet the students’, supervisors’, and organizations’ needs. One participant opined that “The SPP curriculum at the Qatar University is of a good quality. Because dietetics is growing rapidly, the SPP curriculum needs to be updated as per the accreditation guidelines.

### Other factors that contribute to employability

Other factors contributing to employability were individual, environmental/contextual, and cultural factors. Individual factors included students’ performance during the SPP (reflected on by supervisors’ evaluations), students’ performance during the job interview, applicants’ previous experience, and grade point average. The environmental/contextual factors were the job and market requirements. Further, prior work experience in a health profession is regarded as an important value for employability in dietitics, as mentioned by eight preceptors in this study.

## Discussion

This study investigated the impact of an IDAS-guided SPP program on students’ skills and attributes development and preparation for potential employment, from the perspectives of preceptors who have hiring authority. The interviews of preceptors revealed that working as supervisors in an IDAS -guided SPP makes their work more interesting and challenging due to the demand, as well as opportunities, for professional learning of the preceptors through the SPP program [15]. The preceptors also developed closer relationships with the graduate students, which can be seen as a preparation for further cooperative and collaborative work relations.

The IDAS-guided SPP program also impacts on graduate students including bridging the gap between classroom didactic knowledge and practice; improvement of self-confidence for the workplace; developing the competencies demanded by the profession, such as critical-thinking skills, communication, interviewing, and counselling skills. The findings are aligned with the objectives of the SPP and overall study programs. Also, our findings from this study confirm previous findings in that an SPP highlighting features of work environment will help to a great extent prepare students for their profession [2, 4, 5]. Earlier Barr et al. [1] conducted a survey on 2000 RDs who passed the national registration examination in the USA. These students like our students also went through the ACEND program. Out of four programs (didactic education, SPP, work experience, and continuing education) studied in the Barr et al’s study, they [1] reported that the SPP contributed the most skill development (44.8%). Respondents indicated that SPP was a critical part of dietetics curriculum. Eyler [2] reinforced that the experiential education bridges the class room academic work with the real life. Experiential education prepares students transition from college to work [2]. These sentiments are also echoed by our study participants. This study also revealed that in an international setting in Qatar, SPP students developed skills necessary to function in multidisciplinary and multicultural environments.

Furthermore, the results of our study contribute to the literature on employability in higher education, particularly in regard to employers’ perspectives, which is an area that has required attention [7, 9]. The preceptors in this study suggested that an IDAS -guided SPP contributes to the graduates’ employability in that it reduces the need for orientation and probation periods by giving students familiarity with the work environment and work flow, thus improving their readiness for work. This result sheds light on the field of SPP in the health profession and, on a larger scale, can be used to design internship programs in general for the purpose of employability.

In addition to the umpteen benefits voiced by supervisors/employers of SPP, they also pointed out a few more factors contributing to employability that deserve attention, including individual factors, previous work experience, and cultural competence. This echoes what has been emphasized in health profession education in general [16, 17]. Because of the dynamic features of dietetics practice, the curriculum must be updated to reflect the advances in the field of dietetics.

Our study has a few significant implications and a few limitations. Firstly, the study provides insights into employers’ perspectives of an IDAS -guided SPP regarding not only their own experiences, but more importantly, the impact on graduate students’ development and potential employability. Nevertheless, the results of the study remain provisional due to the limited sample size. Another limitation of this study was lack of graduate employment records. It is important to note that the trainees were not actually employed and attributes and skills they acquired during the SPP may help them obtain an employment. This synthesis was based on supervisors’ perception and interpretation of the impact of their own training of the SPP students. Comparative studies are needed to bring together both preceptors’ and graduate students’ perspectives. In addition, longitudinal studies to observe graduates’ growth in their first-year of work would give more insights into the long-term impact of the SPP program and IDAS influence.

## Conclusion

This study has found that the IDAS -guided SPP provides an up-to-date curriculum and improves the quality of the nutrition and dietetics program. Our findings demonstrate the positive impact of IDAS -guided SPP on the development of overall skills and attributes of dietetics students prior to transitioning to the workforce. Dietetic graduates that have a battery of practical skills gained during SPP are better equipped to deliver safe and effective nutrition care at various employment platforms. We recommend that international universities outside the USA with active dietetics/nutrition programs should consider developing their curriculum based on the IDAS competencies for a didactic dietetics program, developing an SPP program as part of either a coordinated program or a stand-alone post-baccalaureate dietetic internship, seek accreditation from IDAS, and institute a nationwide registration/licensure examination for registering dietetic professionals as registered dietitians.

## Data Availability

All data are generated in this study are included in this published article.
